# Clinical features of osteoporosis in patients with type 1 and type 2 diabetes in Türkiye: A nationwide study

**DOI:** 10.1007/s11657-026-01677-x

**Published:** 2026-02-24

**Authors:** Dilek Gogas Yavuz, Cem Haymana, Tolga Akkan, Muhiddin Yalcin, Zeliha Hekimsoy, Ilker Tasci, Mehmet Ali Eren, Naim Ata, Suayip Birinci, Alper Sonmez, Fahri Bayram

**Affiliations:** 1 Section of Endocrinology and Metabolism, Marmara University School of Medicine, Istanbul, Türkiye; 2 Department of Endocrinology and Metabolism, University of Health Sciences, Gulhane School of Medicine, Ankara, Türkiye; 3 Department of Endocrinology and Metabolism, University of Health Sciences, Eskişehir City Hospital, Ankara, Türkiye; 4 Department of Endocrinology and Metabolism, Gazi University School of Medicine, Ankara, Türkiye; 5 School of Medicine, Department of Endocrinology and Metabolism, Celal Bayar University, Manisa, Türkiye; 6 School of Medicine, Department of Geriatrics, Ufuk University, Ankara, Türkiye; 7 School of Medicine, Department of Endocrinology and Metabolism, Lokman Hekim University, Ankara, Türkiye; 8 Department of Strategy Development, Turkish Ministry of Health, Ankara, Türkiye; 9 Deputy Minister of Health, Turkish Ministry of Health, Ankara, Türkiye; 10 Department of Endocrinology and Metabolism, Ankara Guven Hospital, Ankara, Türkiye; 11 Department of Endocrinology and Metabolism, Erciyes University School of Medicine, Kayseri, Türkiye

**Keywords:** Osteoporosis, Type 1 Diabetes, Type 2 Diabetes, Fragility Fractures, Complications Macrovascular, Complications Microvascular, Nationwide Study

## Abstract

***Summary*:**

This nationwide study assessed clinical features of osteoporosis in Turkish adults with diabetes. Osteoporosis prevalence was 9.36% in type 1 diabetes and 19.13% in type 2 diabetes, with higher rates in women and older individuals. Although overall fracture prevalence was low, fractures were markedly more frequent in patients with osteoporosis. Despite the high burden, most patients did not receive osteoporosis therapy, and age, female sex, and vascular complications were independently associated with osteoporosis.

**Aim:**

Fragility fractures related to diabetes are increasing worldwide with rising life expectancy. We aimed to investigate the prevalence of osteoporosis and its associated factors in a large nationwide cohort of adults with type 1 (T1DM) and type 2 diabetes (T2DM) in Türkiye.

**Methods:**

Using Turkish Ministry of Health electronic health records (2015–2021), adults with T1DM and T2DM were identified and classified according to the presence or absence of osteoporosis. Demographics, comorbidities, diabetes-related complications, fracture diagnoses, and laboratory parameters were analyzed. Factors independently associated with osteoporosis were evaluated using multivariable logistic regression.

**Results:**

The cohort included 76,742 individuals with T1DM (mean age 44.3 ± 19.1 years; 45.8% women) and 7,006,910 with T2DM (mean age 59.1 ± 13.4 years; 60.2% women). Overall osteoporosis prevalence was 19.03% and was 9.3% in T1DM and 19.1% in T2DM. Osteoporosis was more frequent in women than men (T1DM: 14.17% vs 5.29% p < 0.001; T2DM: 27.76% vs 6.04% p < 0.001) and increased with age in both diabetes types. Fractures occurred in 0.22% of patients with T1DM and 0.21% of those with T2DM. In T1DM, fracture frequency was approximately sixfold higher in patients with osteoporosis than in those without osteoporosis (RR ≈ 6.92; 95% CI 5.09–9.39; p < 0.001), whereas in T2DM osteoporosis was associated with an approximately fourfold higher fracture frequency (RR ≈ 3.98). Macrovascular and microvascular complications were more frequent among osteoporosis patients with T1DM than those with T2DM. Only 13.4% of patients with osteoporosis received pharmacological osteoporosis therapy. In multivariable analysis, older age (OR 1.06), female sex (OR 6.58), cardiovascular disease (OR 1.58), and microvascular complications (OR 1.38) were independently associated with osteoporosis.

**Conclusion:**

In this nationwide Turkish cohort, osteoporosis affected 9.36% of adults with T1DM and 19.13% of those with T2DM. Among patients with osteoporosis, vascular complications were more prevalent in T1DM than in T2DM.Despite the substantial disease burden and the markedly higher fracture frequency in individuals with osteoporosis, most patients remained untreated.

**Supplementary Information:**

The online version contains supplementary material available at 10.1007/s11657-026-01677-x.

## Introduction

Both diabetes and osteoporosis are increasingly prevalent diseases, in part owing to aging populations worldwide. There is an increased risk of fracture (particularly marked at the hip) in patients with type 1 diabetes and type 2 diabetes, which is less concordant with bone mineral density (BMD) decrement than the general population, implying that there are other factors that contribute to increased fracture risk [1]. Diabetes-induced osteoporosis represents the combined impact of conventional osteoporosis with the additional fracture burden attributed to diabetes [2].

Studies have found a relative risk (RR) of hip fractures of 4.93 in people with type 1 diabetes and a RR of 1.33 in people with type 2 diabetes compared to people without diabetes [3]. The mechanisms of increased fracture risk in diabetes are multifaceted. Type 1 diabetes is typically associated with reduced BMD, whereas it is not usually observed in type 2 diabetes. [4] However, both types of diabetes can impact bone health through various pathways, including impaired bone formation, increased marrow adiposity, and compromised bone matrix quality caused by hyperglycemia [4]. Increased risk of fractures in diabetes has been linked to various clinical factors, including disease duration, poor glycemic control, presence of vascular complications, and medications [3, 4]. Furthermore, complications of diabetes, such as retinopathy and autonomic dysfunction, may contribute to bone fractures by increasing the risk of falls [5]. On the other hand, identifying and managing fracture risk in individuals with diabetes remains a significant challenge. Conventional tools for fracture risk assessment, such as BMD and Fracture Risk Assessment Tool scores, may underestimate the risk in these patients [2].

The prevalence of osteoporosis and diabetes-fracture association may vary across different populations and be influenced by race and ethnicity [5]. Osteoporosis is consistently more prevalent in individuals with type 2 diabetes across global populations, ranging from 15–38%, depending on age, sex, and method of assessment [6–11]. Investigation of the frequency and determinants of osteoporosis in individuals with type 1 and type 2 diabetes in Türkiye can provide valuable insights that could potentially inform targeted interventions and healthcare policies for this at-risk population. This study aimed to investigate the frequency and associated factors of osteoporosis in a large, nationwide sample of individuals with type 1 and type 2 diabetes in Türkiye.

## Methods

### Study design and subjects

This study utilized the Turkish Ministry of Health National electronic database. In this retrospective observational study, data were obtained from the Turkish national electronic health record infrastructure, a unified system used across priamry secondary and tertiary public healthcare institutions in Türkiye. This platform integrates standardized patient medical records and laboratory results and provides nationwide coverage, as health data are centrally collected and maintained under the governance of the Turkish Ministry of Health. The system is also linked to the national electronic prescription (e-prescription) database, enabling comprehensive capture of medication use in routine clinical practice.

Patients with a diagnosis of type 1 and type 2 diabetes were identified using the International Classification of Diseases—10 (ICD-10) codes and Anatomical Therapeutic Chemical (ATC) Classification codes (see below) from January 2015 through December 2021 [Supplementary Figure S1]. The study was conducted according to the ethical norms and standards in the Declaration of Helsinki. The Ministry of Health's Central Ethical Board approved the study protocol (approval date and number: 27 November 2019/95741,342–020).

### Data collection and definitions

Diagnosis and classification of diabetes based on American diabetes association criteria (2). Type 1 diabetes was screened using the ICD-10 code E10 and its extensions and concomitant prescription of insulin (ATC code A10AB, A10AC, A10AD, A10AE) but no oral antidiabetic drug (ATC code A10B) prescription between the specified dates [Supplementary Figure S1]. Incorrect or inappropriate ICD codes were identified and removed during data cleaning prior to analysis.

The first recorded diabetes diagnosis within the 2015–2021 screening period was considered the index date. Osteoporosis and other comorbidities were identified based on the first occurrence of the corresponding ICD codes recorded in conjunction with diabetes. For laboratory parameters, the most recent available measurements were used for analysis.

Patients not compatible with type 1 diabetes were recorded as type 2 diabetes. Osteoporosis was screened using the ICD-10 codes M80 and M81 [Supplementary Table S1]. ICD-10 codes used for the identification of concomitant diseases were as follows: obesity, E66; hypertension, I10; dyslipidemia, E78; heart failure, I50.9; coronary artery disease, I25.1; peripheral artery disease, I73.9; and cerebrovascular disease, I67. Microvascular complications were screened using H36 and E11.2 ICD-10 codes.

Fracture diagnoses recorded using ICD codes were based on radiological evaluation, including X-ray, CT, and MRI findings. Trauma-induced fractures were excluded. Osteoporosis was diagnosed according to World Health Organization criteria, based on bone mineral density measurements: a T-score ≤ − 2.5 in postmenopausal women or men aged ≥ 55 years, a Z-score ≤ − 2.0 in premenopausal women or younger men, and/or the presence of a fragility fracture.(15).

Osteoporosis-related fractures were identified using the S71.87, S32, and S52.8 ICD-10 codes.. Hip and knee prostheses were screened with Z96.64 and Z96.65 ICD-10 codes. Anti-osteoporotic medications and calcium and vitamin D supplements were recorded from the database using the ATC codes [Supplementary Table S2].

Demographic and clinical characteristics at the last visit, including age, sex, comorbidities, diabetic complications, anti-diabetic medications, and body mass index (BMI), were recorded. BMI was calculated as the ratio of weight to height squared (kg/m2).

Comorbid diseases included obesity, dyslipidemia, hypertension, coronary artery disease, heart failure, cerebrovascular disease, peripheral artery disease, chronic obstructive pulmonary disease (COPD), and hypothyroidism. Chronic kidney disease (CKD), retinopathy, and peripheral neuropathy were recorded as the diabetes complications.

Laboratory records included HbA1c, low-density lipoprotein (LDL)-cholesterol, creatinine, calcium, phosphate, parathormone (PTH) and 25OH vitamin D levels. The estimated glomerular filtration rate (eGFR) was calculated using the CKD-EPI equation. Osteoporotic fractures were classified as vertebral, hip, and forearm. Hip and knee prosthesis data were also recorded.

Obesity was defined as a BMI ≥ 30 kg/m^2^. CKD was specified as eGFR < 60 mL/min/1.73 m^2^ using the CKD-EPI equation.

Patients died before 2021 and with end stage renal disease with e GFR 15 ml//min/1.73 m^2^ were excluded from the study.

### Statistical analysis

Statistical analysis was performed in SPSS 18.0 (SPSS Inc., Chicago, IL, USA). Data were expressed as mean and standard deviation or median and interquartile range (25th-75th) for continuous variables or as number of cases and percentages (%) for categorical variables.

Comparisons between patients with type 1 diabetes and type 2 diabetes, as well as between those with and without osteoporosis, were performed using the Mann–Whitney U test for continuous variables and the chi-square test or Fisher’s exact test for categorical variables, as appropriate.

Binary logistic regression was performed to ascertain the association between different variables and the presence of osteoporosis in patients with type 1 and type 2 diabetes. The inclusion of variables in the model was contingent upon two criteria: statistical significance (p < 0.05) in univariate analysis and clinical rationale indicating a potential association with osteoporosis. The selected variables included sex, age, atherosclerotic cardiovascular diseases (coronary artery disease, heart failure, stroke or peripheral artery disease), any microvascular complications (diabetic kidney disease, retinopathy or neuropathy), and the type of diabetes. Variables were evaluated at a 95% confidence level, and a value of p < 0.05 was considered statistically significant.

## Results

The national diabetes cohort consisted of 76,742 patients (mean age: 44.3 ± 19.1 years, women: 45.5%) with type 1 diabetes and 7,006,910 patients with type 2 diabetes (mean age: 59.1 ± 13.4 years, women: 60.2%). Osteoporosis was observed in 9,3% (n = 7183) of patients with type 1 diabetes, and 19.1% (n = 7,006,910) of patients with type 2 diabetes. Demographic characteristics, comorbidities, and diabetic complications among patients with and without a diagnosis of osteoporosis in T1DM and T2DM groups are shown in Table 1. T1DM and T2Dm groups demographic,clinica and laboratory data are shown in Supplementary Table S3.

**Table 1:** Demographic, clinical, and laboratory characteristics of T1DM and T2DM groups according to osteoporosis status

	Type 1 Diabetes (n = 76,742)					Type 2 Diabetes (n = 7,006,910)					p^§^
	n	No osteoporosis (n = 69,559)	n	Osteoporosis (n = 7,183)	p	n	No osteoporosis (n = 5,666,325)	n	Osteoporosis (n = 1,340,585)	p	
Demographics											
Age, years – median (IQR)	69,559	37 (26)	7,183	67 (23)	< 0.001	5,666,325	58 (17)	1,340,585	67 (14)	< 0.001	< 0.001
Sex, female – n (%)	69,559	30,170 (43.37%)	7,183	4,981 (69.34%)	< 0.001	5,666,325	3,051,858 (53.86%)	1,340,585	1,172,612 (87.47%)	< 0.001	< 0.001
BMI, kg/m^2^ – median (IQR)	6,208	25.24 (6.75)	701	27.55 (7.03)	< 0.001	442,282	30.02 (6.68)	115,654	30.09 (6.62)	0.172	< 0.001
Comorbid diseases											
Hypertension – n (%)	69,559	33,357 (47.95%)	7,183	6,262 (87.18%)	< 0.001	5,666,325	4,223,334 (74.53%)	1,340,585	1,226,028 (91.45%)	< 0.001	< 0.001
Obesity – n (%)	6,208	1,261 (20.31%)	701	227 (32.38%)	< 0.001	442,282	221,439 (50.07%)	115,654	58,744 (50.79%)	< 0.001	< 0.001
Dyslipidemia – n (%)	69,559	36,123 (51.93%)	7,183	5,204 (72.45%)	< 0.001	5,666,325	3,897,440 (68.78%)	1,340,585	1,052,212 (78.49%)	< 0.001	< 0.001
Hypothyroidism – n (%)	69,559	15,426 (22.18%)	7,183	2,244 (31.24%)	< 0.001	5,666,325	1,310,511 (23.13%)	1,340,585	463,451 (34.57%)	< 0.001	< 0.001
Complications											
Coronary artery disease – n (%)	69,559	16,050 (23.07%)	7,183	4,242 (59.06%)	< 0.001	5,666,325	2,147,185 (37.89%)	1,340,585	727,500 (54.27%)	< 0.001	< 0.001
Stroke – n (%)	69,559	1,971 (2.83%)	7,183	608 (8.46%)	< 0.001	5,666,325	180,814 (3.19%)	1,340,585	75,574 (5.64%)	< 0.001	< 0.001
Peripheral artery disease – n (%)	69,559	5,249 (7.55%)	7,183	1,259 (17.53%)	< 0.001	5,666,325	408,621 (7.21%)	1,340,585	149,648 (11.16%)	< 0.001	< 0.001
Heart failure – n (%)	69,559	5,695 (8.19%)	7,183	1,867 (25.99%)	< 0.001	5,666,325	506,655 (8.94%)	1,340,585	200,936 (14.99%)	< 0.001	< 0.001
ASCVD – n (%)	69,559	18,598 (26.74%)	7,183	4691 (65.31%)	< 0.001	5,666,325	2,365,884 (41.75%)	1,340,585	798,885 (59.59%)	< 0.001	< 0.001
Chronic kidney disease – n (%)	42,952	7,193 (16.75%)	5,232	2,167 (41.42%)	< 0.001	3,433,533	485,291 (14.13%)	949,584	226,223 (23.82%)	< 0.001	< 0.001
Retinopathy – n (%)	69,559	5,175 (7.44%)	7,183	837 (11.65%)	< 0.001	5,666,325	154,469 (2.73%)	1,340,585	47,445 (3.54%)	< 0.001	< 0.001
Neuropathy – n (%)	69,559	16,920 (24.32%)	7,183	3,241 (45.12%)	< 0.001	5,666,325	1,177,864 (20.79%)	1,340,585	457,879 (34.16%)	< 0.001	< 0.001
Any microvascular complications – n (%)	69,559	23,618 (33.95%)	7,183	4,513 (62.83%)	< 0.001	5,666,325	1,575,246 (27.8%)	1,340,585	603,277 (45%)	< 0.001	< 0.001
Fractures and prostheses											
Vertebral fracture – n (%)	69,559	39 (0.06%)	7,183	40 (0.56%)	< 0.001	5,666,325	4,031 (0.07%)	1,340,585	4,303 (0.32%)	< 0.001	< 0.001
Hip fracture – n (%)	69,559	48 (0.07%)	7,183	29 (0.4%)	< 0.001	5,666,325	3,171 (0.06%)	1,340,585	2733 (0.2%)	< 0.001	< 0.001
Forearm fracture – n (%)	69,559	11 (0.02%)	7,183	1 (0.01%)	0.689	5,666,325	528 (0.01%)	1,340,585	247 (0.02%)	< 0.001	0.619
Hip prosthesis – n (%)	69,559	5 (0.01%)	7,183	3 (0.04%)	0.032	5,666,325	460 (0.01%)	1,340,585	262 (0.02%)	< 0.001	0.169
Knee prosthesis – n (%)	69,559	10 (0.01%)	7,183	3 (0.04%)	0.115	5,666,325	2484 (0.04%)	1,340,585	1681 (0.13%)	< 0.001	0.042
Laboratory tests											
HbA1c, % – median (IQR)	27,099	8.45 (2.9)	2,934	7.8 (2.46)	< 0.001	2,077,519	6.9 (2.45)	572,274	6.7 (1.85)	< 0.001	< 0.001
LDL-C, mg/dl – median (IQR)	30,137	104 (45.8)	3,866	105 (49.65)	0.701	2,613,033	117 (49)	731,374	119.5 (50)	< 0.001	< 0.001
eGFR, mL/min/1.73m^2^ – median (IQR)	42,115	96.6 (39.13)	5,182	67.36 (42.38)	< 0.001	3,419,652	85.09 (29.09)	946,961	76.26 (29.06)	< 0.001	< 0.001
Creatinine, mg/dl – median (IQR)	42,115	0.81 (0.32)	5,182	0.93 (0.51)	< 0.001	3,419,652	0.8 (0.29)	946,961	0.79 (0.28)	< 0.001	< 0.001
Calcium, mg/dl – median (IQR)	5,996	9.4 (0.74)	341	9.22 (0.8)	< 0.001	551,059	9.45 (0.7)	80,815	9.49 (0.7)	< 0.001	< 0.001
Phosphorus, mg/dl – median (IQR)	4,410	3.5 (0.97)	279	3.52 (0.92)	0.829	421,054	3.5 (0.8)	84,418	3.6 (0.8)	< 0.001	0.1
25-OH vitamin D, ng/dl – median (IQR)	12,848	16 (13.8)	1,863	19.27 (16.6)	< 0.001	943,588	16.77 (13.68)	347,957	20.25 (16.1)	< 0.001	< 0.001
iPTH, pg/dl – median (IQR)	3,449	42 (37.95)	542	53.08 (63.95)	< 0.001	186,858	48.8 (36.85)	79,735	50.54 (38.75)	< 0.001	0.004

^**§**^*Comparison of type 1 and type 2 diabetes in patients with osteoporosis*

### Osteoporosis prevalence by age and sex

Patients with osteoporosis were older than those without osteoporosis in both type 1 diabetes (66 vs. 37 years) and type 2 diabetes (67 vs. 58 years). Among osteoporotic patients, those with type 1 diabetes were younger than those with type 2 diabetes (66 vs. 67 years).

**Fig. 1:**
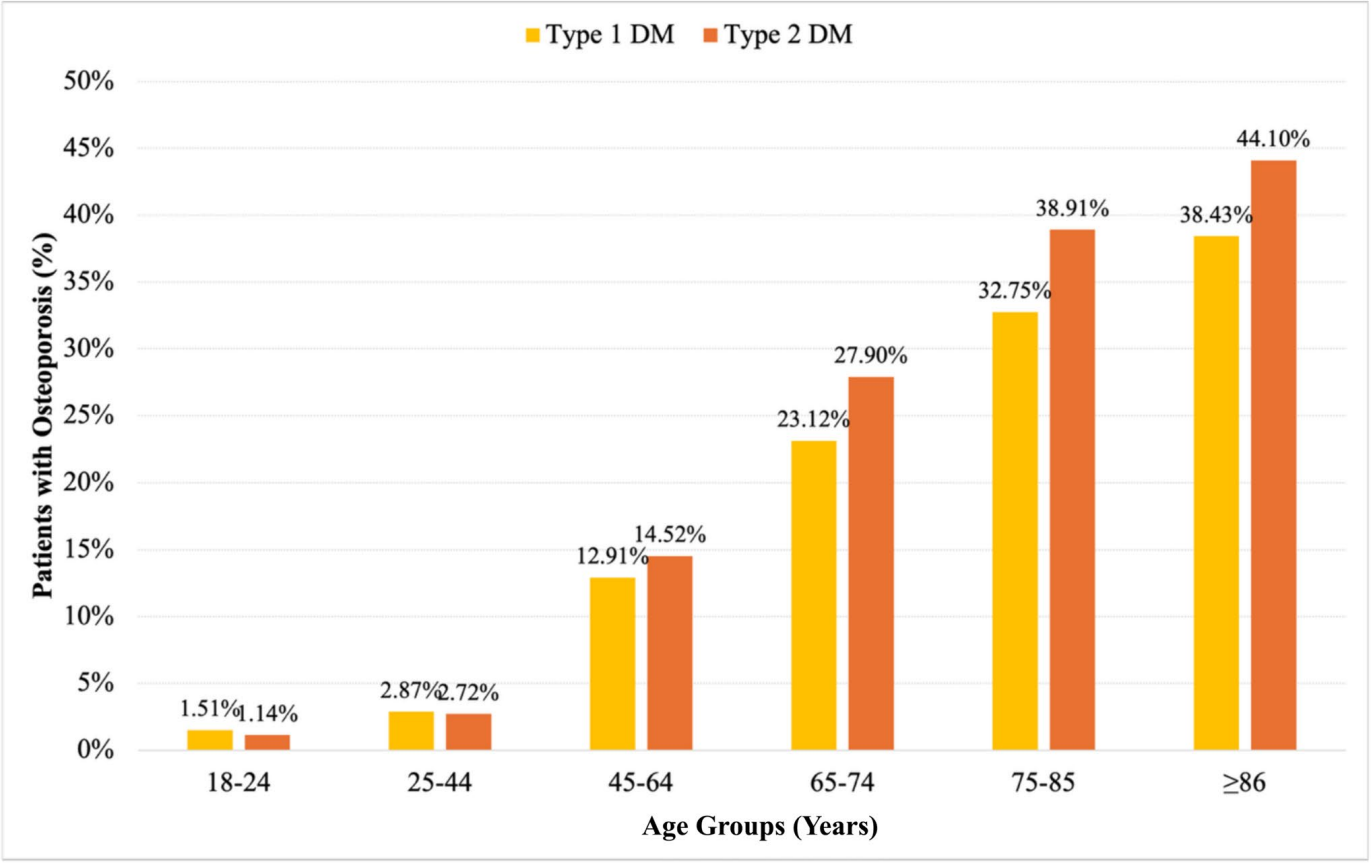
Distribution of patients with type 1 and type 2 diabetes diagnosed with osteoporosis according to age groups

The age-based distribution of osteoporosis diagnoses among patients with type 1 diabetes was as follows: 18–24 years: 205 patients (1.51%), 25–44 years: 959 patients (2.87%), 45–64 years: 1,948 patients (12.91%), 65–74 years: 1,926 patients (23.12%), 75–85 years: 1,793 patients (32.75%), > 85 years: 352 patients (38.43%). The age-based distribution of osteoporosis diagnoses among patients with type 2 diabetes was as follows: 18–24 years: 858 patients (1.14%), 25–44 years: 24,826 patients (2,72%), 45–64 years: 501,179 patients (14.52%), 65–74 years: 482,795 patients (27.9%), 75–85 years: 281,657 patients (38.91%), > 85 years: 49,270 patients (44.1%) (Fig. 1).

Osteoporosis was identified in *men* with type 1 diabetes and type 2 diabetes by 5.9% (n = 2.202) and 6,04% (n = 1.340.585), respectively. Women with type 1 diabetes and women with type 2 diabetes were found to have an osteoporosis diagnosis by 14.7% (n = 4.981) and %27.7 (n = 1.172.612), respectively. A higher proportion of women vs. men in the osteoporosis group was evident in both type 1 (69,34% vs. 30,6%) and type 2 diabetes (87,4% vs. 53.8%).

Overall prevalence was 0.12% for vertebral fractures, 0.08% for hip fractures, and 0.01% for forearm fractures in the total cohort. In type 1 diabetes, the total number of reported fractures (vertebral + hip + forearm) was 168, corresponding to an overall fracture prevalence of 0.22%. In type 2 diabetes, 15,013 fractures were recorded, yielding a similar overall prevalence of 0.21%. Within the type 1 diabetes group, fracture prevalence was 0.14% in patients without osteoporosis compared with 0.97% in those with osteoporosis, indicating an approximately 6.9-fold higher fracture frequency in the presence of osteoporosis (RR ≈ 6.92; 95% CI 5.09–9.39; p < 0.001). Similarly, in type 2 diabetes, fracture prevalence was 0.136% among patients without osteoporosis and 0.543% in those with osteoporosis, corresponding to an approximately fourfold higher fracture frequency associated with osteoporosis (RR ≈ 3.98).

### Comorbidities and diabetes complications in patients with and without osteoporosis

Mean BMI in type 1 diabetes and type 2 diabetes patients with and without osteoporosis were 27.5 kg**/m**^**2**^vs 25.2 kg**/m**^**2**^ and 30.09 kg**/m**^**2**^vs 30.02 kg**/m**^**2**^ respectively (p < 0.001). Hypertension, dyslipidemia, and hypothyroidism were significantly higher in patients with osteoporosis than in those without osteoporosis in patients with both types of diabetes. The prevalence of obesity was significantly higher in patients with osteoporosis T1DM group, while there was no between-group difference in T2DM group.

The prevalence of major complications in patients with osteoporosis with type 1 diabetes and type 2 diabetes were as follows; coronary artery disease: 59.06% and 54.27%, stroke: 8.46% and 5.64%, peripheral artery disease: 17.53% and 11.16%, heart failure: 25.99% and 14.99%, chronic kidney disease: 41.42% and 23.82%, retinopathy: 11.65% and 3.54%, and neuropathy: 45.12% and 34.16%.

### Biochemical findings in patients with and without osteoporosis

Patients with type 1 diabetes with and without an osteoporosis diagnosis had a median (IQR) HbA1c level of 7.8% (2.46) and 8.45% (2.9), respectively. Similarly, patients with type 2 diabetes with and without an osteoporosis diagnosis had a median (IQR) HbA1c level of 6.7% (1.85) and 6.9% (2.45), respectively. The median LDL-cholesterol levels were slightly higher in patients with osteoporosis in the two diabetes groups. The median eGFR level was lower in patients with osteoporosis compared with non-osteoporotic individuals. The median calcium levels were slightly lower in patients with osteoporosis and type 1 diabetes, and slightly higher in patients with osteoporosis and type 2 diabetes compared to non-osteoporotic patients. Phosphorus levels were similar in patients with type 1 diabetes with and without an osteoporosis diagnosis, however slightly higher in patients with osteoporosis and type 2 diabetes compared to non-osteoporotic patients with type 2 diabetes.

Patients with both type 1 and type 2 diabetes and osteoporosis had higher mean 25-OH vitamin D levels than those without osteoporosis. Mean 25-OH vitamin D levels were below 20 ng/dL in patients with type 1 diabetes and osteoporosis and without osteoporosis. Patients with type 2 diabetes and osteoporosis had a mean 25-OH vitamin D level of 20.2 ng/dL.

Mean iPTH levels were near the upper limit of the normal range in non-osteoporotic patients (44.80 pg/mL), while osteoporotic patients had higher iPTH levels (61.30 pg/mL) within the normal range. Osteoporotic and non-osteoporotic patients with type 2 diabetes had similar mean iPTH levels. (Table 1).

### Renal Function, Diabetes Treatment, and Anti-Osteoporotic Medication Use

In both type 1 and type 2 diabetes groups, patients with osteoporosis consistently demonstrated poorer renal function based on eGFR. Diabetes treatment pattern did not differ between patients with and without osteoporosis (Table 2).

**Table 2:** Renal function, antidiabetic therapy, and antiosteoporotic treatment characteristics of the study groups

	Type 1 Diabetes (n = 76,742)					Type 2 Diabetes (n = 7,006,910)					p^§^
	n	No osteoporosis (n = 69,559)	n	Osteoporosis (n = 7,183)	p	n	No osteoporosis (n = 5,666,325)	n	Osteoporosis (n = 1,340,585)	p	
Renal function	42,115		5,182		< 0.001	3,419,652		946,961		< 0.001	< 0.001
eGFR ≥ 90 mL/min/1.73m^2^ – median (IQR)		24,953 (59.25%)		1,264 (24.39%)	< 0.001		1,395,441 (40.81%)		246,100 (25.99%)	< 0.001	0.099
eGFR 60–89 mL/min/1.73m^2^ – median (IQR)		11,131 (26.43%)		1,817 (35.06%)	< 0.001		1,586,568 (46.4%)		485,453 (51.26%)	< 0.001	< 0.001
eGFR 45–59 mL/min/1.73m^2^ – median (IQR)		2,853 (6.77%)		935 (18.04%)	< 0.001		299,280 (8.75%)		142,929 (15.09%)	< 0.001	< 0.001
eGFR 30–44 mL/min/1.73m^2^ – median (IQR)		2,092 (4.97%)		736 (14.2%)	< 0.001		111,528 (3.26%)		59,663 (6.3%)	< 0.001	< 0.001
eGFR 15–29 mL/min/1.73m^2^ – median (IQR)		1,086 (2.58%)		430 (8.3%)	< 0.001		26,835 (0.78%)		12,816 (1.35%)	< 0.001	< 0.001
Antidiabetic treatment						5,666,325		1,340,585		< 0.001	
1 oral antidiabetic – n (%)							2,199,259 (38.81%)		546,859 (40.79%)	< 0.001	
2 oral antidiabetics – n (%)							958,336 (16.91%)		220,736 (16.47%)	< 0.001	
≥ 3 oral antidiabetics – n (%)							734,660 (12.97%)		174,621 (13.03%)	0.062	
Insulin – n (%)							1,061,765 (18.74%)		256,186 (19.11%)	< 0.001	
Osteoporosis treatment											
Alendronate – n (%)			7,183	150 (2.09%)				1,340,585	29,778 (2.22%)		0.468
Ibandronate – n (%)			7,183	102 (1.42%)				1,340,585	29,523 (2.2%)		< 0.001
Risedronate – n (%)			7,183	16 (0.22%)				1,340,585	4,934 (0.37%)		0.046
Zoledronate – n (%)			7,183	74 (1.03%)				1,340,585	16,801 (1.25%)		0.102
Denosumab – n (%)			7,183	99 (1.38%)				1,340,585	16,321 (1.22%)		0.213
Teriparatide – n (%)			7,183	2 (0.03%)				1,340,585	302 (0.02%)		0.679
Oral calcium supplements – n (%)	69,559	842 (1.21%)	7,183	1,083 (15.08%)	< 0.001	5,666,325	101,829 (1.8%)	1,340,585	221,132 (16.5%)	< 0.001	0.001
Oral cholecalciferol supplements – n (%)	69,559	12,910 (18.56%)	7,183	2399 (33.4%)	< 0.001	5,666,325	1,323,921 (23.36%)	1,340,585	538,020 (40.13%)	< 0.001	< 0.001

^**§**^*Comparison of type 1 and type 2 diabetes in patients with osteoporosis*

Only 6.17% of type 1 diabetic patients with osteoporosis and 7.28% of type 2 diabetic patients with osteoporosis were prescribed an antiosteoporosis medication. Alendronate (2.09%) was the most frequently prescribed drug in patients with type 1 diabetes and osteoporosis. Alendronate and Ibandronate were the most frequently and equally prescribed drugs in patients with type 2 and osteoporosis. Denosumab prescription was slightly higher in patients with type 1 diabetes then type 2 diabetes, whereas Zoledronate prescription was not different between the groups. (Table 2).

### The independent predictors of an osteoporosis diagnosis

**Fig. 2:**
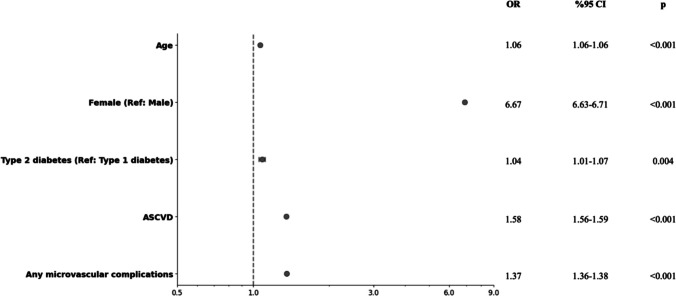
Multivariate logistic regression analysis in order to determine the independent predictors of an osteoporosis diagnosis

In the multivariate regression analysis, age (OR: 1.06, CI: 1.06–1.06, p < 0.001), female sex (OR: 6.67, CI: 6.63–6.71, p < 0.001), type 2 diabetes (OR: 1.04, CI: 1.01–1.07, p = 0.004), atherosclerotic cardiovascular disease (OR: 1.58, CI: 1.56–1.59, p < 0.001) and any microvascular complications (OR: 1.37 CI: 1.36–1.38, p < 0.001) showed a direct association with an osteoporosis diagnosis (Fig. 2).

## Discussion

This study found osteoporosis in 9.3% of patients with type 1 diabetes and 19.1% of patients with type 2 diabetes in Türkiye. These findings imply that Türkiye's osteoporosis burden is high, but it is lower than in many Middle Eastern and Asian populations and closer to European type 2 diabetes studies. Patients with type 2 diabetes in the Middle East had osteoporosis at 29.4% in Saudi Arabia and 55.7% in older Iranian women [9, 10] in previous studies. Osteoporosis rates range from 27.7% in China to 35.5% to 49.5% in India [7, 11]. A recent pooled analysis demonstrated a 27.67% prevalence of osteoporosis in patients with type 2 diabetes [12]. A European data analysis showed 13.0% of men and 21.9% of women with type 2 diabetes had osteoporosis [13].

Differences in study methods, population demographics, genetic variables, lifestyle impacts, healthcare access, and diagnostic techniques may collectively explain variations in osteoporosis prevalence. Therefore, assessment of regional numbers and population-specific risk variables is crucial in achieving prevention and treatment goals in osteoporosis in people with diabetes.

As expected, people with osteoporosis in the present dataset were older than those without in both type 1 and type 2 diabetes. On the other hand, patients with type 1 diabetes and osteoporosis were younger than patients with type 2 diabetes and osteoporosis (37 vs. 58 years), possibly due to disease-related bone loss. Our study also confirmed the age-associated increase in osteoporosis diagnoses in both type 1 and type 2 diabetes populations. In the type 1 diabetes group, osteoporosis prevalence increased from 1.5% in 18–24 year old category to 38.4% in those aged ≥ 85 years. In the type 2 diabetes cohort, prevalence rose from 1.1% in 18–24 years to 44.1% in ≥ 85 years. Previous studies showed that aging is a major risk factor for osteoporosis in patients with diabetes. Leidig-Bruckner et al. reported a direct correlation between age and osteoporosis diagnosis in patients with type 1 diabetes, particularly in older adults [17]. Concerning type 2 diabetes, older adults, particularly women, have a higher risk of osteoporosis related to age and gender [18, 19].

The prevalence of osteoporosis in men with type 1 diabetes (5.9%) and type 2 diabetes (6.0%) was similar, consistent with previous studies showing minimal risk differences. However, postmenopausal women with type 2 diabetes have a higher risk of osteoporosis due to obesity and metabolic complications, as demonstrated by the 27.7% higher prevalence of osteoporosis compared with type 1 diabetes (14.7%) (6,14). There were more women in both type 1 (69,34% vs. 30,6%) and type 2 diabetes (87,4% vs. 53.8%). groups, which is in agreement with the predominance of female sex in osteoporosis diagnoses.

We identified that BMI was slightly higher in patients with either type 1 diabetes and type 2 diabetes and an osteoporosis diagnosis. Some studies have implied that a greater BMI was protective against fracture risk owing to mechanical loading, while others suggested an augmented bone fragility because of obesity-related inflammation [15]. On the other hand, patients with type 1 diabetes and osteoporosis are at a higher fracture risk (0.92%) than patients with type 2 diabetes (0.55%) due to lower peak bone mass and insulin deficiency–related bone microarchitecture changes [6].

Due to aging and metabolic illness burden, osteoporotic individuals live with more comorbidities than the normal population [8, 16]. In the current study, patients with type 1 diabetes and osteoporosis diagnosis had higher rates of cardiovascular and microvascular complications like coronary artery disease, stroke, peripheral artery disease, heart failure, CKD, retinopathy, and neuropathy than patients with type 2 diabetes. Longer disease duration and earlier vascular involvement may explain the difference in comorbidity burden between the two types of diabetes [6].

These findings underscore that diabetic osteoporosis risk is multifaceted and that age, sex, comorbidities, and diabetes type should be considered when assessing fracture risk and implementing preventative interventions. Moreover, longer disease duration, higher body mass index, and comorbidities that harm bone health may explain why type 2 diabetes has a higher prevalence of osteoporosis across all age groups than type 1. These findings are in agreement with the previously reported link between type 2 diabetes and fractures due to poor bone quality, despite normal or higher BMD [1, 2, 14].

In the current study, osteoporosis patients had worse renal function than non-osteoporotic patients in both diabetes types, but it was more evident in patients with type 1 diabetes. Dialysis dependency was higher in type 1 diabetics with osteoporosis (14.7%) than those without (4.8%). Also, patients with an eGFR < 15 mL/min/1.73 m^2^ were more than twice as likely to be osteoporotic (9.6%) compared to non-osteoporotic (4.1%). These data suggest a link between advanced CKD and osteoporosis in type 1 diabetes, possibly due to metabolic changes like insulin shortage and extended disease duration that might affect bone and kidney functions. Moreover, type 1 diabetes is associated with earlier and more severe CKD-MBD, which increases skeletal fragility and fracture risk [20]. Although the type 2 diabetes and osteoporosis combination showed lower renal function, the difference was less evident. Dialysis-dependency was 0.6% in the osteoporotic group and 0.4% in the non-osteoporotic group, whereas those with an eGFR < 15 mL/min/1.73 m^2^ were 0.5% and 0.4%, respectively. It should be noted that the relationship between CKD and osteoporosis in type 2 diabetes may be masked by other risk factors like age, obesity, and sedentary lifestyle [21]. In the current study, the persistent relationship between CKD and osteoporosis emphasizes the necessity for integrated bone health and renal function care in diabetics, especially in patients with type 1 diabetes.

We found higher 25-OH vitamin D blood levels in patients with type 1 and type 2 diabetes mellitus and osteoporosis than in the group without osteoporosis. Although this observation was unexpected, past studies have shown that patients with osteoporosis more frequently take vitamin D supplementation [22, 23], which may explain the higher 25-OH vitamin D blood levels in our study population. On the other hand, the mean 25-OH vitamin D level in patients with type 2 diabetes and osteoporosis was still well below the recommended level (≥ 30 ng/mL) for bone health [24].

This study found very low rates of anti-osteoporotic drug prescriptions among patients with diabetes mellitus and osteoporosis. With only 6.2% of patients with type 1 diabetes and 7.3% of those with type 2 diabetes receiving any therapy, the treatment rates may be the lowest in the literature so far. Indeed, in contrast to achievements in screening and diagnosing, starting medical treatment for osteoporosis remains low. Of the European women at high fracture risk, only 29% are treated properly [25]. Among Japanese patients admitted to the hospital with an osteoporotic fracture, 91.7% reported no previous treatment, while only 25.5% were treated during hospitalization [26]. Over the past decade, US osteoporosis treatment rates have dropped from 15 to 8%, a worrying trend that is worse among patients with diabetes [27]. The poor treatment rates in type 1 and type 2 diabetes emphasize the need for increased awareness, screening, and proactive care to reduce diabetes-related fracture risk. These findings indicate the necessity for integrated treatment methods that include early osteoporosis diagnosis and adequate pharmaceutical medication in diabetic populations due to the known links between diabetes, bone fragility, and fracture risk [28].

## Conclusion

This study found that osteoporosis prevalence rises with age in patients with both types of diabetes, with type 2 diabetes consistently having higher rates across all age groups in Türkiye. Osteoporosis is linked to a higher burden of macrovascular and microvascular complications in both types of diabetes, highlighting the complex nature of skeletal fragility in these populations. Despite this increased risk, anti-osteoporotic therapy was found to be very low, revealing a true gap in treatment in disease management. These findings highlight the need for early screening, preventative methods, and suitable pharmacologic therapies to minimize fracture risk and osteoporosis-related morbidity in type 1 and type 2 diabetics.

## Supplementary Information

Below is the link to the electronic supplementary material.

Supplementary file1 (DOCX 212 KB)
