# Supplementary material for: Clinical features of osteoporosis in patients with type 1 and type 2 diabetes in Türkiye: A nationwide study
Source: Arch Osteoporos. 2026 Feb 24;21(1):41. doi: 10.1007/s11657-026-01677-x (PMC12929295; doi:10.1007/s11657-026-01677-x)
Supplement: Supplementary file 1 — Supplementary file1 (DOCX 212 KB) [file 11657_2026_1677_MOESM1_ESM.docx]

**Supplementary Material**

**Title**

CLINICAL FEATURES OF OSTEOPOROSIS IN TYPE 1 AND TYPE 2 DIABETIC PATIENTS IN TÜRKIYE: A LARGE SCALE, NATIONWIDE STUDY

**Authors**

Dilek Gogas Yavuz, Cem Haymana, Tolga Akkan, Muhiddin Yalçin, Zeliha Hekimsoy, Ilker Tasci, Mehmet Ali Eren, Naim Ata, Suayip Birinci, Alper Sönmez, Fahri Bayram

**Table of contents**

**Supplemantary Figure S1:** Definitions of type 1 and type 2 diabetes mellitus.

**Supplementary Table S1:** Distribution of provinces in Türkiye in order of socioeconomic status.

**Supplementary Table S2.** ATC codes of anti-osteoporotic medications and calcium and vitamin D supplements.

**Supplementary Figure S1. Definitions of type 1 and type 2 diabetes mellitus.**


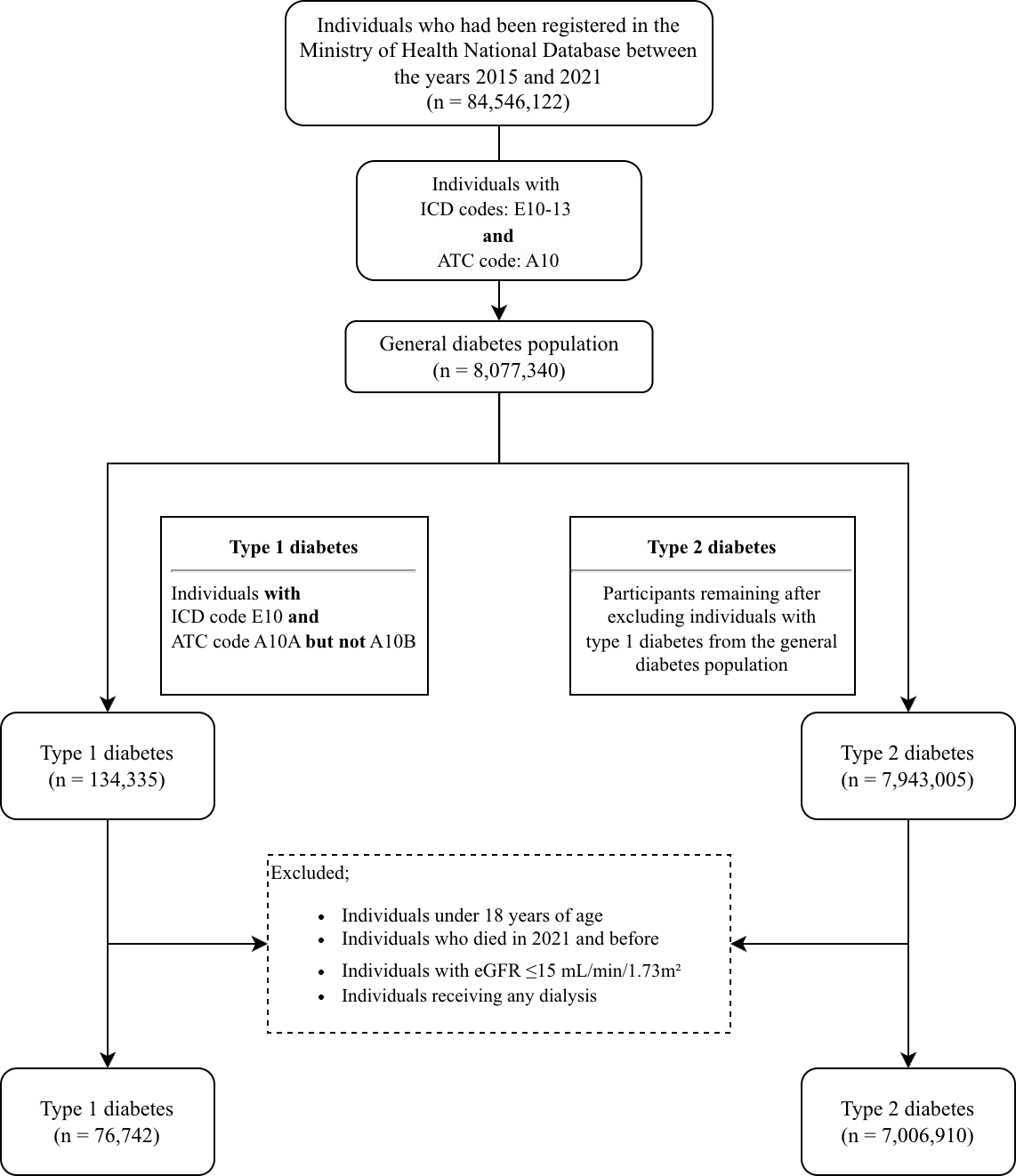


**ICD; International Statistical Classification of Diseases and Related Health Problems, ATC;* *Anatomical Therapeutic Chemical Classification, eGFR; estimated glomerular filtration rate.*

**Supplementary Table S1. Definitions of osteoporosis, complications and comorbidities of diabetes.**

| Disease | Definition |
| --- | --- |
| Osteoporosis | - ICD-10 code: M80, M81 and subsections - ATC code: M05B, H05A |
| Hypertension | - ICD-10 code: I10 - ATC code: C02 |
| Obesity | - BMI ≥30 kg/m^2^ - ICD-10 code: E66 and subsections |
| Dyslipidemia | - ICD-10 code: E78 and subsections - LDL-C ≥ 100 mg/dL - Triglyceride ≥ 150 mg/dL |
| Hypothyroidism | - ICD-10 code: E03 and subsections - ATC code: H03A |
| Coronary artery disease | - ICD-10 code: I21, I22, I25 and subsections - HID code: Coronary artery bypass graft operation (604910, 604920, 604930, 604940, 604950, 604960, 604970, 604980, 604990, 605000, 605010), therapeutical cardiac catheterization (700880, 700890, 700900, 700910, 700920, 700921, 700922) |
| Heart failure | - ICD-10 code: I50 and subsections |
| Stroke | - ICD-10 code: I63, I69 and subsections |
| Peripheral artery disease | - ICD-10 code: I73.8 and I73.9 - HID code: Amputation procedure (611220, 611230, 611240, 611250, 611860, 909880, 910480), hyperbaric oxygen treatment (702580, 702590) - ATC code: B01AC11 (iliomedin) |
| Chronic kidney disease | - eGFR: <60 mL/min/1.73 m² - Albumin-to-creatinine ratio ≥30 mg/g - ICD-10 code: N18 and subsections - HID code: Hemodialysis (704220, 704230), peritoneal dialysis (704231) |
| Retinopathy | - ICD-10 code: E10.3, E11.3, E13.3, E14.3 - HID code: Laser treatment for retinopathy (617630) - ATC code: L01XC07 (bevacuzimab) |
| Neuropathy | - ICD code: G63.2 |

**ICD; International Statistical Classification of Diseases and Related Health Problems, ATC;* *Anatomical Therapeutic Chemical Classification, BMI; Body mass index, LDL-C; low density lipoprotein cholesterol, HID; Health Implementation Decleration.*

**Supplementary Table S2. ATC codes of anti-osteoporotic medications and calcium and vitamin D supplements.**

| Medications | ATC code |
| --- | --- |
| Alendronate | M05BB03, M05BA04 |
| Ibandronate | M05BA06 |
| Risedronate | M05BB07, M05BA07 |
| Zoledronic acid | M05BA08 |
| Denosumab | M05BX04 |
| Teriparatide | H05AA02 |
| Calcium carbonate | A12AA04 |
| Calcium carbonate + vitamin D3 | A12AX |
| Vitamin D3 | A11CC05 |

**Supplementary Table S3: Demographic, clinical, and laboratory characteristics of patients with type 1 and type 2 diabetes**

|  | **All participants**  **(n=7,083,652)** | | | | |
| --- | --- | --- | --- | --- | --- |
|  | **n** | **Type 1 DM**  **(n = 76,742)** | **n** | **Type 2 DM**  **(n = 7,006,910)** | **p** |
| **Demographics** |  | | | | |
| Age, years – median (IQR) | 76,742 | 39 (30) | 7,006,910 | 60 (17) | <0.001 |
| Sex, female – n (%) | 76,742 | 35,151 (45.8%) | 7,006,910 | 4,224,470 (60.29%) | <0.001 |
| BMI, kg/m^2^ – median (IQR) | 6,909 | 25.46 (6.96) | 557,936 | 30.04 (6.68) | <0.001 |
| **Comorbid diseases** |  | | | | |
| Hypertension – n (%) | 76,742 | 39,619 (51.63%) | 7,006,910 | 5,449,362 (77.77%) | <0.001 |
| Obesity – n (%) | 6,909 | 1,488 (21.54%) | 557,936 | 280,183 (50.22%) | <0.001 |
| Dyslipidemia – n (%) | 76,742 | 41,327 (53.85%) | 7,006,910 | 4,949,652 (70.64%) | <0.001 |
| Hypothyroidism – n (%) | 76,742 | 17,670 (23.03%) | 7,006,910 | 1,773,962 (25.32%) | <0.001 |
| Osteoporosis – n (%) | 76,742 | 7,183 (9.36%) | 7,006,910 | 1,340,585 (19.13%) | <0.001 |
| **Complications** |  | | | | |
| Coronary artery disease – n (%) | 76,742 | 20,292 (26.44%) | 7,006,910 | 2,874,685 (41.03%) | <0.001 |
| Stroke – n (%) | 76,742 | 2,579 (3.36%) | 7,006,910 | 256,388 (3.66%) | <0.001 |
| Peripheral artery disease – n (%) | 76,742 | 6,508 (8.48%) | 7,006,910 | 558,269 (7.97%) | <0.001 |
| Heart failure – n (%) | 76,742 | 7,562 (9.85%) | 7,006,910 | 707,591 (10.1%) | 0.025 |
| ASCVD – n (%) | 76,742 | 23,289 (30.35%) | 7,006,910 | 3,164,769 (45.17%) | <0.001 |
| Chronic kidney disease – n (%) | 48,184 | 9,360 (19.43%) | 4,383,117 | 711,514 (16.23%) | <0.001 |
| Retinopathy – n (%) | 76,742 | 6,012 (7.83%) | 7,006,910 | 201,914 (2.88%) | <0.001 |
| Neuropathy – n (%) | 76,742 | 20,161 (26.27%) | 7,006,910 | 1,635,743 (23.34%) | <0.001 |
| Any microvascular complications – n (%) | 76,742 | 28,131 (36.66%) | 7,006,910 | 2,178,523 (31,09%) | <0.001 |
| **Fractures and prostheses** |  | | | | |
| Vertebral fracture – n (%) | 76,742 | 79 (0.1%) | 7,006,910 | 8,334 (0.12%) | 0.201 |
| Hip fracture – n (%) | 76,742 | 77 (0.1%) | 7,006,910 | 5,904 (0.08%) | 0.127 |
| Forearm fracture – n (%) | 76,742 | 12 (0.02%) | 7,006,910 | 775 (0.01%) | 0.232 |
| Hip prosthesis – n (%) | 76,742 | 8 (0.01%) | 7,006,910 | 722 (0.01%) | 0.974 |
| Knee prosthesis – n (%) | 76,742 | 13 (0.02%) | 7,006,910 | 4,165 (0.06%) | <0.001 |
| **Laboratory tests** |  | | | | |
| HbA1c, % – median (IQR) | 30,033 | 8.4 (2.89) | 2,649,793 | 6.81 (2.3) | <0.001 |
| LDL-C, mg/dl – median (IQR) | 34,003 | 104 (46.3) | 3,344,407 | 117.32 (50) | <0.001 |
| eGFR, mL/min/1.73m² – median (IQR) | 47,297 | 94.16 (41.81) | 4,366,613 | 83.21 (29.56) | <0.001 |
| Creatinine, mg/dl – median (IQR) | 47,297 | 0.82 (0.33) | 4,366,613 | 0.80 (0.28) | <0.001 |
| Calcium, mg/dl – median (IQR) | 6,337 | 9.4 (0.73) | 631,874 | 9.45 (0.7) | <0.001 |
| Phosphorus, mg/dl – median (IQR) | 4,689 | 3.5 (0.97) | 505,472 | 3.5 (0.8) | 0.22 |
| 25-OH vitamin D, ng/dl – median (IQR) | 14,711 | 16.39 (14.17) | 1,291,545 | 17.6 (14.56) | <0.001 |
| iPTH, pg/dl – median (IQR) | 3,991 | 43.30 (41.4) | 266,593 | 49.2 (37.4) | <0.001 |
